# Why the binary latent growth model is not a special case of the ordinal latent growth model: Theoretical arguments and empirical evidence

**DOI:** 10.3758/s13428-026-03043-8

**Published:** 2026-05-05

**Authors:** Kyungmin Lim, Su-Young Kim

**Affiliations:** Department of Psychology, Ewha Womans University, Seoul, Republic of Korea

**Keywords:** Binary LGM, Ordinal LGM, Scale assignment, Scaling information, Categorical LGM

## Abstract

In the structural equation modeling framework, binary variable models are generally considered a special case of ordinal variable models, as both involve similar scale assignment processes. However, the scaling processes of the two model types differ, with these differences becoming increasingly pronounced in the context of latent growth models (LGMs). To define scale units, the two types of LGMs—specifically, one with ordinal variables and the other with binary variables—depend on different observed scale references, such as thresholds and standard deviations, which are derived from observed categorical variables. Applying distinct observed scale references to binary and ordinal LGMs results in systematic differences in the scale units of their corresponding latent response variables. Consequently, in binary LGMs, the transformed latent response variables used for model estimation may fail to accurately reflect the corresponding population information, and as a result, their parameter estimates are more likely to be systematically biased than those obtained from ordinal LGMs. This study investigates the impact of these differences on estimating ordinal and binary LGMs and underscores potential estimation concerns in binary LGMs from both theoretical and empirical perspectives.

## Introduction

Latent growth models (LGMs; McArdle, 1986; Meredith & Tisak, 1990) are widely utilized in the social sciences to examine longitudinal changes in individuals’ responses to a given variable across multiple time points. While LGMs have been predominantly applied to continuous observed variables, increasing demand exists for their application to categorical data—such as binary (e.g., “yes” or “no”) and ordinal (e.g., “strongly disagree” to “strongly agree”) responses—resulting in growing interest in LGMs with categorical indicator variables (i.e., categorical LGMs; Simons-Morton et al., 2004; Verhagen & Fox, 2012; Yong et al., 2012). The principles underlying the estimation of categorical LGMs have primarily been discussed in the context of LGMs with ordinal variables (i.e., ordinal LGMs) rather than those with binary variables (i.e., binary LGMs; Grimm & Liu, 2016; Masyn et al., 2013; Mehta et al., 2004; Muthén & Asparouhov, 2002). This is because, in the structural equation modeling framework, models are estimated using latent continuous variables (i.e. latent response variables) that underlie the observed categorical variables, and binary variables are treated as ordinal variables with only two categories (Agresti, 2018; Kamata & Bauer, 2008; Muthén & Asparouhov, 2002).

Indeed, the ordinal and binary models are distinct, as they depend on separate “observed scale references” for scaling the latent response variables. The term “observed scale references” refers to the information derived from observed categorical variables, such as thresholds, standard deviations, and other relevant metrics. For example, in ordinal LGMs, to define the scale of a latent response variable, the observed scale references correspond to the thresholds derived from the proportions of each category. In contrast, for binary LGMs, both the threshold and the standard deviation are used to define the scale of latent response variable, so the observed scale references consist of the threshold and the standard deviation. The differences in the observed scale references applied to ordinal and binary variables have a significant impact on estimation, particularly in the context of LGMs, which cannot be ignored. Consequently, binary LGM estimates are more likely to be distorted than ordinal LGM estimates. That is, using binary data in a categorical LGM may reduce the accuracy and stability of the estimates. This study investigates how differences in the observed scale references applied to ordinal and binary variables impact categorical LGM estimation and, through relevant simulations, examines the negative impact of utilizing binary observed variables on LGM estimation from both theoretical and empirical perspectives.

### Systematic differences in the scaling of latent response variables between ordinal and binary LGMs

Structural equation models (SEMs) with categorical variables (i.e., categorical SEMs) are generally defined as models for latent response variables rather than observed variables; moreover, the parameter estimates are also provided within the context of these latent response variables. This approach is based on the categorical variable methodology (CVM; Muthén, 1984; Muthén & Kaplan, 1985), which posits that an individual’s response to a categorical variable is determined by their underlying continuous latent tendency. According to the CVM, all observed categorical responses can be transformed into underlying latent response variables. These transformed variables do not possess a specific scale or metric, unlike observed variables. Therefore, for model estimation, it is necessary to manually assign scale information, such as measurement origin and unit, to all latent response variables.

To set the scale of these latent response variables, observed scale references are utilized. The observed scale reference reflects the information obtained from the observed sample through categorical variables onto the transformed latent response variables by determining the location of the scale’s origin and the length of the unit. One notable point is that the type of observed scale references used for scale setting varies depending on whether the categorical variable is ordinal or binary. For ordinal variables, two thresholds are used as observed scale references (Jöreskog, 1990; Mehta et al., 2004), with the position of the first threshold being the origin and the interval between the two thresholds determining the unit length. By contrast, binary variables have only a single threshold, making it impossible to apply the same method. Consequently, additional information (e.g., the standard deviation of the binary variable)—beyond that provided by the threshold alone—is required to establish the scale unit (Lim & Kim, 2024).

Applying different observed scale references to binary and ordinal models leads to systematic differences in the scale units of their corresponding latent response variables. In other words, the unit length of the scale may vary depending on which observed scale reference is employed in each model. Notably, in categorical SEMs, the standard deviation of each transformed latent response variable is determined relative to the scale unit applied to that variable. For example, a latent response variable with a smaller unit length exhibits a larger standard deviation than others. Therefore, if the unit lengths of the scales differ between ordinal and binary models, the resulting standard deviations of the latent response variables are also likely to differ across the two models.

In the context of conventional confirmatory factor analysis (CFA), however, there is likely to be no significant difference in the standard deviation values estimated by the two models. This is because categorical CFA typically assumes that all observed variables measuring a single factor will exhibit similar response patterns (Bollen & Lennox, 1991). In such cases, regardless of which observed scale reference is utilized (e.g., the interval between thresholds of an ordinal variable or the standard deviation of a binary variable), the relative scale units of the transformed latent response variables will be similar across items, resulting in comparable standard deviation estimates. For this reason, most studies employing such models do not address in depth the difference in observed scale references between binary and ordinal models (Babakus et al., 1987; Flora & Curran, 2004; Kamata & Bauer, 2008; Rhemtulla et al., 2012). The differences in model estimates observed across these two cases are therefore often attributed not to a systematic cause due to the observed scale references but rather to the fact that categorical variables with more response categories tend to behave more like continuous variables, thereby improving the accuracy of parameter estimation (Agresti, 2018; McCullagh & Nelder, 1989).

In contrast, categorical LGMs, which primarily aim to examine changes in response tendencies over time (Bollen & Curran, 2006; Muthén & Asparouhov, 2002), are expected to demonstrate variations in response patterns across time points. As a result, the scale units of the latent response variables at each time point may vary depending on the observed scale references used, and the standard deviations determined by the relative scale unit lengths may also differ. As such models inherently involve heteroscedasticity across all indicator variables (Lim & Kim, 2024), the standard deviations of the latent response variables must be accurately calculated at each time point to correctly estimate key parameters of interest (e.g., the mean of the slope factor). However, while the standard deviations of latent response variables can be relatively accurately estimated in ordinal LGMs, in binary LGMs the standard deviations of these variables are likely to be incorrectly estimated due to the nature of the observed scale references employed for scaling.

Nevertheless, many studies comparing the estimation performance of ordinal and binary models have predominantly been conducted within the context of CFA (Beauducel & Herzberg, 2006; Dolan, 1994; Ferrari & Barbiero, 2012; Flora & Curran, 2004; Forero et al., 2009; Li, 2016; Rhemtulla et al., 2012). As noted earlier, there are fundamental differences between CFA and LGM in terms of model characteristics, particularly regarding the influence of observed scale references on estimation results. Therefore, conclusions drawn from previous studies on the impact of the number of categories of categorical variables in CFA cannot be directly applied to LGMs. In other words, the differences in estimation performance when using binary and ordinal variables in categorical LGMs should be examined with careful consideration of the unique features of LGMs.

### What is known and what is unknown about the difference between binary and ordinal LGMs

Many methodological studies have examined the relationship between the number of categories and estimation performance in categorical LGMs. Numerous simulation studies have consistently reported that using binary variables can impair estimation (Finch, 2017; Lee et al., 2017; Newsom & Smith, 2020). The critical issue, however, has not been addressed yet: why the use of binary LGMs leads to deterioration in estimation performance. While some studies have empirically compared estimation outcomes between ordinal and binary LGMs, they have provided little theoretical explanation for the observed differences (Finch, 2017; Lee et al., 2017). Most research has either treated binary LGMs as a subtype of ordinal LGMs or focused on differences in degrees of freedom for model identification (Bollen & Curran, 2006; Jöreskog, 1990; Lim & Kim, 2024; Muthén, 1996), without addressing the fundamental distinctions in the scaling process between the two models. Consequently, no in-depth examination of the systematic differences in estimation performance between ordinal and binary LGMs has been conducted. This may lead researchers to attribute the differences solely to the smaller information provided by fewer categories, overlooking limitations inherent to the model itself and underestimating potential challenges in binary LGM estimation.

This study primarily aims to examine the impact of differences in observed scale references on estimation in categorical LGMs, thereby identifying potential estimation issues in binary LGMs from both theoretical and empirical perspectives. To this end, the study first clarifies the fundamental principles by which the distributional characteristics of latent response variables (i.e., means and standard deviations) are determined based on observed scale references in ordinal and binary LGMs. Subsequently, it theoretically elucidates how differences in the standard deviations of latent response variables between the two models affect model estimation. Additionally, it offers empirical evidence regarding estimation issues arising from the use of binary variables in LGMs by comparing the estimation performance of ordinal and binary LGMs through a simulation study.

## Categorical LGM

Categorical LGMs are specified in a manner similar to LGMs with continuous data (i.e., continuous LGMs) using latent response variable ${y}^{*}$, as shown in Eq. 1.

1 $${y}_{it}^{*}={\eta }_{0i}+{\eta }_{1i}{\lambda }_{t}+{e}_{it},$$

where $${y}_{it}^{*}$$ is the latent response tendency for individual $$i$$ at time $$t$$, $${\eta }_{0i}$$ is the intercept of the individual growth trajectory which consists of a mean intercept ($${\alpha }_{00}$$) and a residual ($${\zeta }_{0i}$$; $${\eta }_{0i}={\alpha }_{00}+{\zeta }_{0i}$$), $${\eta }_{1i}$$ is the slope of the individual growth trajectory which contains a mean slope ($${\alpha }_{10}$$) and a residual($${\zeta }_{1i}$$; $${\eta }_{1i}={\alpha }_{10}+{\zeta }_{1i}$$), $${e}_{it}$$ is the measurement error, and $${\lambda }_{t}$$ is the growth factor loading, usually set to ($$t-1)$$ at time $$t$$ for centering the intercept at the first measurement occasion. According to Eq. 1, $${y}^{*}$$ is described as having an individual growth trajectory for each person, characterized by distinct $${\eta }_{0i}$$ and $${\eta }_{1i}$$. The categorical LGM explores the average change in $${y}^{*}$$ over time by modeling a single mean growth trajectory across individual growth trajectories.

Using Eq. 1, the model-implied mean ${\widehat{\mu }}_{t}^{*}$ (Eq. 2), variance ${\widehat{\sigma }}_{t}^{*}$ (Eq. 3), and covariance between two time points $t$ and $u$, ${\widehat{\sigma }}_{tu}^{*}$ (Eq. 4) can be defined as follows:

2 $${\widehat{\mu }}_{t}^{*}={\alpha }_{00}+{\alpha }_{10}{\lambda }_{t},$$

3 $${\widehat{\sigma }}_{t}^{*}=Var\left({\zeta }_{0i}+{\zeta }_{1i}{\lambda }_{t}\right)+Var\left({e}_{it}\right)={\psi }_{00}+2{\lambda }_{t}{\psi }_{10}+{\lambda }_{t}^{2}{\psi }_{11}+{\theta }_{t},$$

4 $${\widehat{\sigma }}_{tu}^{*}={\psi }_{00}+\left({\lambda }_{t}+{\lambda }_{u}\right){\psi }_{10}+{\lambda }_{t}{\lambda }_{u}{\psi }_{11},$$

where ${\psi }_{00}$ is the variance of ${\zeta }_{0i}$, ${\psi }_{11}$ is the variance of ${\zeta }_{1i}$, ${\psi }_{10}$ is the covariance between ${\zeta }_{0i}$ and ${\zeta }_{1i}$, and ${\theta }_{t}$ is the variance of measurement error, ${e}_{it}$. Equation 2 shows that ${\widehat{\mu }}_{t}^{*}$ represents the mean intercept of the individual growth trajectories for all individuals $i$, and that it changes over time by the amount of the mean slope. In other words, the average change in ${y}^{*}$ over time can be observed by estimating ${\alpha }_{00}$ and ${\alpha }_{10}$. Meanwhile, Eq. 3 indicates that, like ${\widehat{\mu }}_{t}^{*}$, ${\widehat{\sigma }}_{t}^{*}$ can also vary over time because of the variance information from ${\zeta }_{1i}{\lambda }_{t}$ and time-specific error variance at each time point. This time-varying heteroscedasticity is one of the defining features of growth models and is considered a key distinction from cross-sectional models (e.g., categorical CFA), which assume homoscedasticity for all latent response variables (Lim & Kim, 2024).

In summary, the categorical LGM is defined as a model that transforms observed categorical variables into latent response variables and utilizes them to explore the changes in latent tendencies (i.e., ${y}^{*}$) over time. However, this model cannot yet be estimated because the ${y}^{*}$ transformed from $y$ does not have a predetermined metric. Therefore, to estimate the model, it is necessary to first establish an appropriate scale for the ${y}^{*}$ variables.

## Common scale construction for categorical LGMs

One critical assumption in scale construction is measurement invariance over time (Millsap & Cham, 2012). In longitudinal models (e.g., categorical LGMs), this assumption should be met because the means of the latent response variables can only be meaningfully compared if measured on the same scale across all time points. To this end, the origin and unit of the scale for all ${y}^{*}$ variables across time points should be identical, thereby establishing a common scale (Mehta et al., 2004). The establishment of such a common scale is regarded as a preliminary step that precedes the selection of parameter constraints used to identify the model and define the final metric of the parameter estimates (Lim & Kim, 2024).

One widely used method is to constrain the thresholds of ${y}^{*}$ to be equal across all time points (Jöreskog, 1994; Masyn et al., 2013). Thresholds (TS) are parameters of the ${y}^{*}$ distribution, representing cutoffs on the latent response tendency at which specific categorical responses occur (Beauducel & Herzberg, 2006). When thresholds are held constant across times, the ${y}^{*}$ variables share the same cutoffs and are, thus, considered as being on the same scale. Based on this logic, thresholds are regarded as parameters that contain information about the scale of the latent response variable, with threshold invariance being employed as evidence for the invariance of the underlying measurement scale of ${y}^{*}$ in categorical LGMs (Bollen & Curran, 2006; Mehta et al., 2004). However, in the case of binary observed variables, satisfying the threshold invariance assumption is not sufficient to establish a common scale for all ${y}^{*}$ variables. While fixing all thresholds across time in ordinal LGMs can ensure the equality of both the origin and unit of the scale, in binary LGMs it only ensures equality of the origin. Therefore, in binary LGMs, additional scale references are required to define the unit of the common scale based on the observed categorical variables. In other words, the threshold invariance assumption cannot serve as a sufficient condition for establishing measurement invariance across both binary and ordinal LGMs.

Given these limitations, it is more appropriate to explain that the common scale required for measurement invariance in categorical LGMs is established not through thresholds, but through observed scale references. As previously mentioned, observed scale references refer to information derived from observed categorical variables that is used to define the origin and unit of the common scale for the latent response variables, with thresholds being just one example of such observed scale references. This section outlines the types of observed scale references employed in ordinal and binary LGMs and explains the principles by which a common scale is established based on these references.

### Observed scale references and principles of common scale setting in ordinal LGMs

In ordinal LGMs, the transformed ${y}^{*}$ is first defined by a standard normal distribution (or standard logistic distribution) at each time point. As the observed response rates change, the ${y}^{*}$ at each time point will have different thresholds on the z-scale. Jöreskog (1994) proposed a method for establishing a common scale and ensuring measurement invariance over time by constraining the thresholds of each ${y}^{*}$ to have the same locations across all time points (i.e., threshold invariance). This assumption is expressed as follows:

5 $${\tau }_{{c}_{1}}={\tau }_{{c}_{2}}=\dots ={\tau }_{{c}_{t}} \;for\; all \;c,$$

where $t$ is the time point. Once the threshold invariance is assumed as in Eq. 5, the locations of any two thresholds can be used as references for defining the origin and unit of the common scale.

**Fig. 1:**
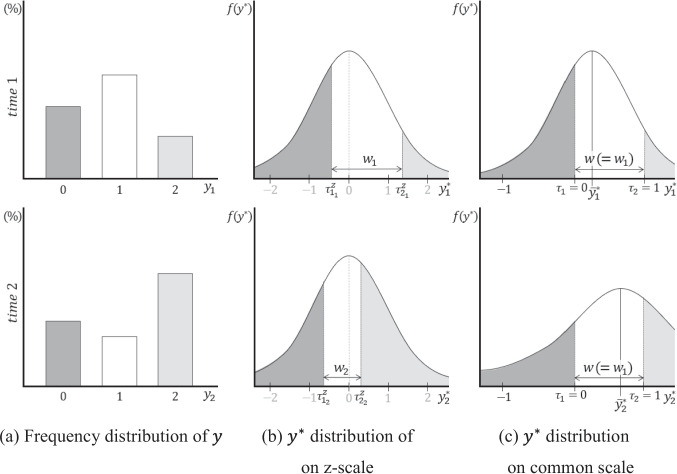
Setting a common scale in ordinal latent growth model using two thresholds as observed scale references. Notes. The notation ${\tau }_{{c}_{t}}^{z}$ is the threshold on the z-scale at each time point, ${\tau }_{c}$ is the threshold on the common scale, ${w}_{t}$ is the unit distance on the z-scale at each time point, $w$ is the unit distance on the common scale, and ${\overline{y} }_{t}^{*}$ is the mean of ${y}^{*}$ on the common scale at each time point

Figure 1 illustrates the principle of setting the common scale using two thresholds for the latent response variable transformed from an observed ordinal variable with three categories measured at two time points ($t=1, 2$). The observed $y$ s (Fig. 1a) are transformed into ${y}^{*}$ s that follow a standard normal distribution (Fig. 1b), with the thresholds ${\tau }_{{1}_{t}}^{z}$ and ${\tau }_{{2}_{t}}^{z}$ on the z-scale being determined by the observed response rates at each time point, respectively. By fixing the locations of ${\tau }_{{1}_{t}}$ and ${\tau }_{{2}_{t}}$ to be 0 and 1, respectively, for all $t$ based on the threshold invariance assumption, the ${y}^{*}$ at each time point has the same origin and unit length (Fig. 1c), and the mean and standard deviation of the distribution are no longer 0 and 1, but different values. This principle of setting the common scale can be applied to all cases where there are two or more thresholds; hence, it is employed in ordinal LGMs with three or more categories for setting the scale of the latent response variable.

### Observed scale references and principles of common scale setting in binary LGMs

In binary LGMs, threshold invariance can also be considered a necessary condition for measurement invariance. However, when the location of a single threshold is fixed across all time points based on the threshold invariance assumption, the ${y}^{*}$ scale at each time point can share the same origin but not the same unit. This is because a binary variable has only one threshold; thus, no information exists regarding the distance between thresholds that can be used as a reference for the unit length of the common scale. As an alternative, in binary LGMs, the standard deviation of the observed binary variable $y$ can be used as an observed scale reference to define the unit length of the ${y}^{*}$ scale following Schweizer’s (2012, 2013) threshold-free approach. One possible suggestion for scaling binary variables was provided by Lim and Kim (2024). This study is significant as it represents the first attempt to clearly explain the scaling of binary LGMs, an issue that has not been explicitly addressed in prior research. However, the set of principles is not only quite complex but also defined by an inverse relationship between the standard deviation of $y$ and that of ${y}^{*}$, potentially rendering it inadequate for fully elucidating the characteristics of ${y}^{*}$ transformed through scaling. Therefore, in the present study, we propose a clearer approach that complements the scaling principles by utilizing the standard deviation of $y$ in setting the common scale of ${y}^{*}$.

**Fig. 2:**
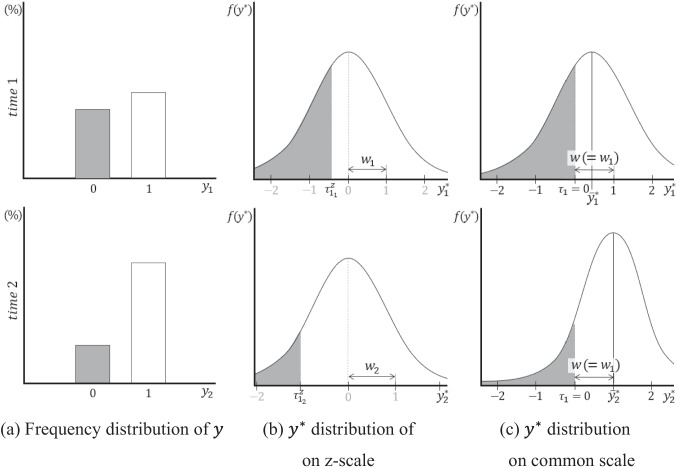
Setting **a** common scale in a binary latent growth model using a single threshold and standard deviation as observed scale references. Notes. The notation ${\tau }_{{c}_{t}}^{z}$ is the threshold on the z-scale at each time point, ${\tau }_{c}$ is the threshold on the common scale, ${w}_{t}$ is the unit distance on the z-scale at each time point, $w$ is the unit distance on the common scale, and ${\overline{y} }_{t}^{*}$ is the mean of ${y}^{*}$ on the common scale at each time point

Figure 2 depicts the setting of the common scale by applying a single threshold and the standard deviation of $y$ as observed scale references in a binary LGM. The response rates for the observed binary variable $y$ at each time point $t$ (Fig. 2a) not only determine the location of the threshold ${\tau }_{{1}_{t}}^{z}$, which is the scale origin of ${y}^{*}$ on the z-scale at each time point (Fig. 2b), but also determine the standard deviation of $y$. According to Schweizer (2013), the distribution of $y$ can be linked to the distribution of ${y}^{*}$ on the z-scale through multiplication by a weight defined as the inverse of the standard deviation. This suggests that the weight is set as the unit length of the scale of ${y}^{*}$ on the z-scale. That is, in the binary LGM, the unit length ${w}_{t}$ of the transformed ${y}^{*}$ at each time point can be defined as follows:

6 $${w}_{t}=\sqrt{\frac{1}{{p}_{t}\left(1-{p}_{t}\right)},}$$

where ${p}_{t}$ is the probability that the individual’s response is 1 at time $t$, and $\sqrt{{p}_{t}(1-{p}_{t})}$ is part of the formula for the standard deviation of $y$ (i.e., $\sqrt{{s}_{t}}=\sqrt{n{p}_{t}\left(1-{p}_{t}\right)}$). The unit distance, ${w}_{t}$, represents the length of one unit of the scale applied to ${y}^{*}$ at each time point, similar to the interval of two thresholds in the ordinal case.

All ${y}^{*}$ s with different unit lengths are assumed to follow a standard normal distribution, as mentioned earlier. In other words, when $y$ at each time point has different standard deviations, the scale of the transformed ${y}^{*}$ that follows a standard normal distribution has different magnitudes of one unit (i.e., ${w}_{t}$) at each time point. Therefore, to establish a common scale, all ${w}_{t}$ values across time points must be set equal, as in the ordinal case. This can be achieved by adjusting the ${w}_{t}$ values of all ${y}^{*}$ s based on that of a specific time point—typically, ${w}_{1}$ (Fig. 2c). Meanwhile, by fixing all ${\tau }_{1}$ to 0 under the threshold invariance assumption, all ${y}^{*}$ s across time points can share the same origin of the scale. In summary, ${\tau }_{1}$ provides the origin of the common scale, and the standard deviation of ${y}_{1}$ provides information about the unit length $w$ of the common scale.

## Distributional differences in latent response variables by number of categories in categorical LGMs

Once an appropriate common scale is assigned to all latent variables, researchers can calculate the distributional characteristics of the transformed ${y}^{*}$ at each time point, namely the sample mean, ${\overline{y} }_{t}^{*}$, and the sample standard deviation, $\sqrt{{s}_{t}^{*}}$.^1^ These distributional characteristics correspond to the model-implied estimates ${\widehat{\mu }}_{t}^{*}$ and $\sqrt{{\widehat{\sigma }}_{t}^{*}}$ of the categorical LGM mentioned earlier, and are used to estimate all growth factor parameters (Eqs. 2 and 3). Nonetheless, such distributional characteristics can vary depending on the observed scale references used to establish the common scale. In this section, we explain the basic principle by which ${\overline{y} }_{t}^{*}$ and $\sqrt{{s}_{t}^{*}}$ are derived through the setting of a common scale in categorical LGMs, and discuss the distributional differences in the transformed ${y}^{*}$ when using binary versus ordinal observed variables.

### Distributional characteristics of latent response variables on the common scale

In ordinal and binary LGMs, once a common scale is established based on appropriate observed scale references, all ${y}^{*}$ s across time points are adjusted as variables on the common scale. As a result, the distribution of ${y}^{*}$ is transformed from the standard normal distribution to normal distributions with different standard deviations (Fig. 1c and 2c). For ordinal and binary categorical LGMs, $\sqrt{{s}_{t}^{*}}$ is determined using the unit length ${w}_{t}$ on the z-scale at each time point as follows:

7 $${w}_{1}\sqrt{{s}_{1}^{*}}={w}_{2}\sqrt{{s}_{2}^{*}}=\dots ={w}_{t}\sqrt{{s}_{t}^{*}}=w,$$

where $\sqrt{{s}_{t}^{*}}$ is the standard deviation of ${y}^{*}$ at time *t* after the common scale is applied. Equation 7 indicates that the standard deviations of ${y}^{*}$ on the common scale are adjusted by the relative magnitude of the unit on the z-scale. The adjusted standard deviation at each time point determines the sample mean of ${y}^{*}$, ${\overline{y} }_{t}^{*}$, at that time point, as follows (Jöreskog, 1994):

8 $${\overline{y} }_{1}^{*}+\sqrt{{s}_{1}^{*}}\times {\tau }_{{c}_{1}}^{z}={\overline{y} }_{t}^{*}+\sqrt{{s}_{t}^{*}}\times {\tau }_{{c}_{t}}^{z},$$

where ${\overline{y} }_{t}^{*}$ is the mean of ${y}^{*}$ at time $t$ on the common scale. Equation 8 demonstrates how ${\overline{y} }_{t}^{*}$ on the common scale at each time point is computed using $\sqrt{{s}_{t}^{*}}$. These sample means and standard deviations influence the model-implied estimates—namely, the mean ${\widehat{\mu }}_{t}^{*}$ and standard deviation $\sqrt{{\widehat{\sigma }}_{t}^{*}}$—and are, ultimately, used to estimate all model parameters.

### Differences in distributions of latent response variables defined in ordinal and binary LGMs

**Fig. 3:**
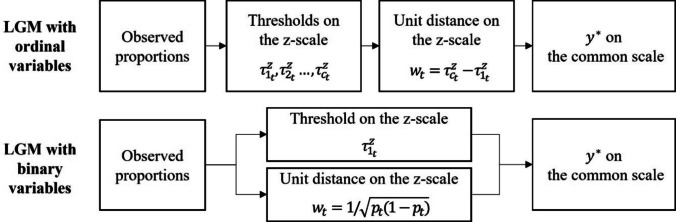
Process of setting ${\tau }_{{c}_{t}}^{z}$ and ${w}_{t}$ based on observed scale references in categorical latent growth model

As indicated in Eqs. 7 and 8, the mean and standard deviation of ${y}^{*}$ on the common scale at each time point are computed using the threshold (i.e., ${\tau }_{{c}_{t}}^{z}$) and the unit length (i.e., ${w}_{t}$) of ${y}^{*}$ on the z-scale, both of which are defined based on the chosen observed scale references. Among these, the size of ${w}_{t}$ at each time point plays a key role in determining both the means and the standard deviations of all ${y}^{*}$ s. One noteworthy point is that, as illustrated in Fig. 3, the order in which ${w}_{t}$ is determined differs between ordinal and binary LGMs because of the use of different observed scale references. Figure 3 illustrates that, in ordinal LGMs, ${\tau }_{{c}_{t}}^{z}$ and ${w}_{t}$ are specified through a sequential process, whereas in binary LGMs, ${\tau }_{{c}_{t}}^{z}$ and ${w}_{t}$ are simultaneously determined based on the observed response proportions.

This difference leads to a clearly distinguishable characteristic in the scale information of the two cases, particularly with respect to ${w}_{t}$. The ${w}_{t}$ at each time point can be determined independently of ${\tau }_{{1}_{t}}^{z}$ in the ordinal LGM, whereas this is not the case in the binary LGM. In the ordinal LGM, because all thresholds are estimated first and then ${w}_{t}$ is determined, ${w}_{t}$ can take on different values depending on the location of ${\tau }_{{2}_{t}}^{z}$, regardless of the value of ${\tau }_{{1}_{t}}^{z}$. For example, if the response proportion of the first category in an ordinal variable is 70%, the scale origin of ${y}^{*}$ on the z-scale would be set to ${\tau }_{{1}_{t}}^{z}$ of 0.52. In this case, the value of ${w}_{t}$ can vary depending on the location of ${\tau }_{{2}_{t}}^{z}$. Therefore, when using ordinal variables, the scale unit carries information that is independent of the origin (as is typical of numerous measurement scales), and its relative value is determined by the interval between the thresholds of all variables. In contrast, in the binary LGM, ${w}_{t}$ and ${\tau }_{{1}_{t}}^{z}$ are both determined directly from the response proportion, and therefore the value of ${w}_{t}$ depends on ${\tau }_{{1}_{t}}^{z}$. For example, in a binary variable with a 70% response rate for the first category (i.e., ${\tau }_{{1}_{t}}^{z}=0.52$), the unit length ${w}_{t}$ is invariably 4.76 (Eq. 6), and no alternative values of ${w}_{t}$ are possible for ${y}^{*}$ on the z-scale with this origin. Ultimately, for binary variables, the unit length is determined as a function of the location of the scale origin. This value is the smallest when the origin is at 0 and increases systematically as the origin moves further away from 0.

**Fig. 4:**
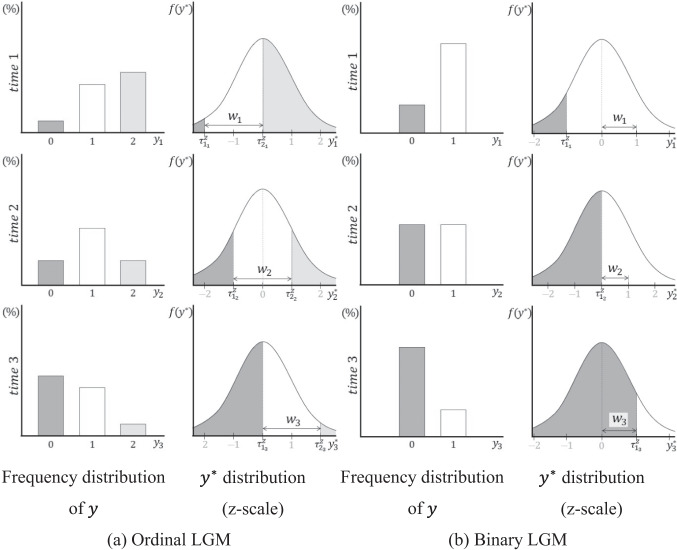
Patterns of response proportions over time for ordinal and binary variables and corresponding ${w}_{t}$ set on ${y}^{*}$ on the z-scale at each time point

Considering the longitudinal nature of the data, where changes in the observed response rates over time are expected (i.e., changes in ${\tau }_{{c}_{t}}^{z}$ over time are expected), the distinctive characteristics of ${w}_{t}$ between ordinal and binary LGMs have a significant impact on determining the distribution of ${y}^{*}$. For example, as illustrated in Fig. 4, when the response rates at three time points are observed as shown on the left, the unit length ${w}_{t}$ at each time point is set according to the observed scale references, as shown on the right, in both the ordinal LGM (Fig. 4a) and the binary LGM (Fig. 4b). Since the overall $\tau$ change on the z-scale is the same in both cases (i.e., $({\tau }_{{1}_{1}}^{z}+{\tau }_{{2}_{1}}^{z})/2=-1$, $({\tau }_{{1}_{2}}^{z}+{\tau }_{{2}_{2}}^{z})/2=0$, and $({\tau }_{{1}_{3}}^{z}+{\tau }_{{2}_{3}}^{z})/2=1$ in the ordinal LGM; ${\tau }_{{1}_{1}}^{z}=-1$, ${\tau }_{{1}_{2}}^{z}=0$, and ${\tau }_{{1}_{3}}^{z}=1$ in the binary LGM), the level of change in the response rates over time can be considered the same. In the case of the ordinal LGM, as illustrated in Fig. 4a, if all intervals between the thresholds of ${y}^{*}$ on the z-scale are equal, the unit length ${w}_{t}$ will also be the same across all time points. Moreover, $\sqrt{{s}_{t}^{*}}$, derived from ${w}_{t}$, will be identical at each time point. In the case of the binary LGM, however, the values of ${w}_{t}$ at the three time points necessarily differ due to changes in response rates over time, as illustrated in Fig. 4b. Therefore, $\sqrt{{s}_{t}^{*}}$ will be the same at the first and third time points but will be larger at the second time point because the unit length is smaller at that time. As a result, even when the observed response rates change at the same level over time, the values of $\sqrt{{s}_{t}^{*}}$ at each time point differ between the ordinal and binary LGMs. ${\overline{y} }_{t}^{*}$, calculated using these standard deviations, will also differ across the two models (Eq. 8). In sum, the differences in the observed scale references applied in each case influence the transformed ${y}^{*}$ distributions employed for model estimation.

## Impact of differences in the latent response variable distribution, determined by number of categories, on categorical LGM estimation

The distributional characteristics of ${y}^{*}$ used for parameter estimation in categorical LGMs can differ between ordinal and binary LGMs. This suggests that the estimation results for categorical LGMs may critically vary depending on the number of categories in the observed variable. Importantly, however, these estimation differences should not be interpreted as radical differences in the underlying population distribution of ${y}^{*}$. The logic of the CVM applied to the estimation of categorical LGMs aligns with the psychometric perspective in the social sciences, which posits that an individual’s responses to certain variables or behavioral outcomes convey their motivation, values, and intellectual capacity (Bandura, 1986; Cohen & Swerdlik, 2017; Deci & Ryan, 1991). From this perspective, the latent tendency that each individual holds toward a given construct is theoretically better understood not as being derived from the observed response, but rather as a factor that precedes and determines it. In other words, ${y}^{*}$ is not a product derived from $y$ but a more fundamental piece of information that exists at the population level and governs the observed $y$. This relationship between $y$ and ${y}^{*}$ has key implications for model estimation using categorical variables. Specifically, regardless of how many categories $y$ has, responses to the same construct can be expected to originate from the same underlying latent variable ${y}^{*}$. For example, when assessing an individual’s level of depression, whether the researcher employs a binary scale or an ordinal Likert-type scale, the individual’s latent level of depression should not differ only because of the type of response scale.

**Fig. 5:**
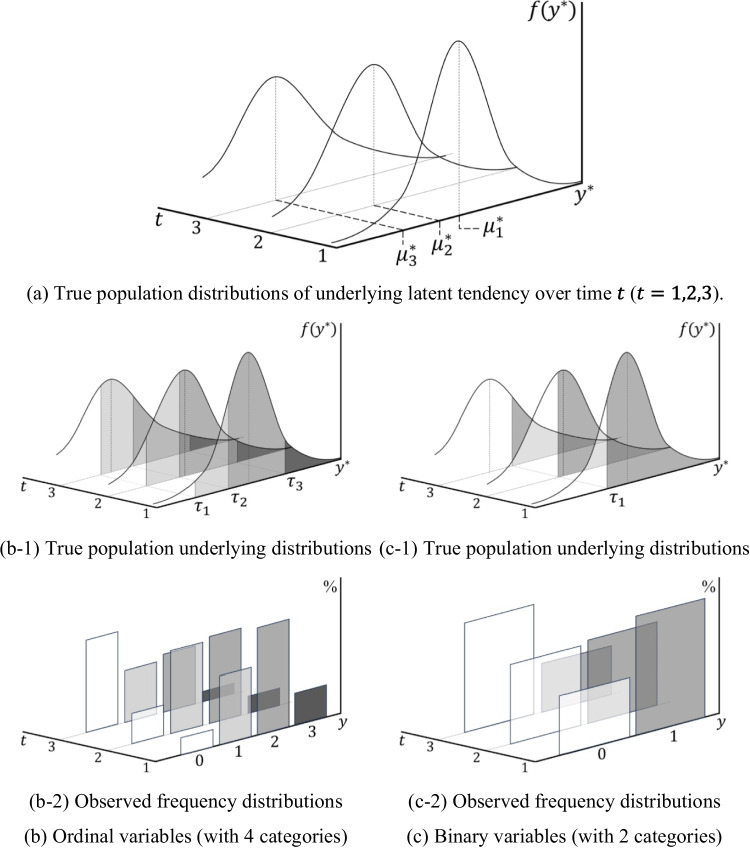
Principle of observing the frequency distribution of $y$ at each time point when using ordinal and binary variables for the same population-level underlying ${y}^{*}$ distribution

Further, the relationship between the population-level latent variable ${y}^{*}$ and the observed sample variable $y$ can be visually represented. Figure 5 illustrates the process of deriving the response frequency distributions for each observed variable $y$ measured using a four-point Likert scale (which includes two negative response categories, 0 = “Strongly Disagree” and 1 = “Disagree,” and two positive response categories, 2 = “Agree” and 3 = “Strongly Agree”) as well as a binary scale with two response categories (negative: 0 = “Disagree” and positive: 1 = “Agree”) for the same underlying ${y}^{*}$. When the true population distribution of the underlying latent tendency for the variable of interest changes over time (Fig. 5a), and the researcher uses an ordinal observed variable with four categories (Fig. 5b), there will be three threshold parameters (${\tau }_{1}$, ${\tau }_{2}$, and ${\tau }_{3}$) for ${y}^{*}$ (Fig. 5b-1). Accordingly, the researcher can observe the frequency distribution of $y$ across the four categories (Fig. 5b-2). On the contrary, if the researcher employs a binary observed variable (Fig. 5c), there will only be one threshold parameter (${\tau }_{1}$) for ${y}^{*}$ (Fig. 5c-1), and the researcher can observe the frequency distribution of $y$ across the two categories (Fig. 5c-2). Importantly, the observed samples in both cases are generated based on the same underlying distribution, differing only in the number of thresholds. This suggests that, regardless of the number of categories in the observed variable used in a categorical LGM, the estimation results for the distribution parameters of ${y}^{*}$ (i.e., ${\mu }_{t}^{*}$ and $\sqrt{{\sigma }_{t}^{*}}$) should be similar.

**Fig. 6:**
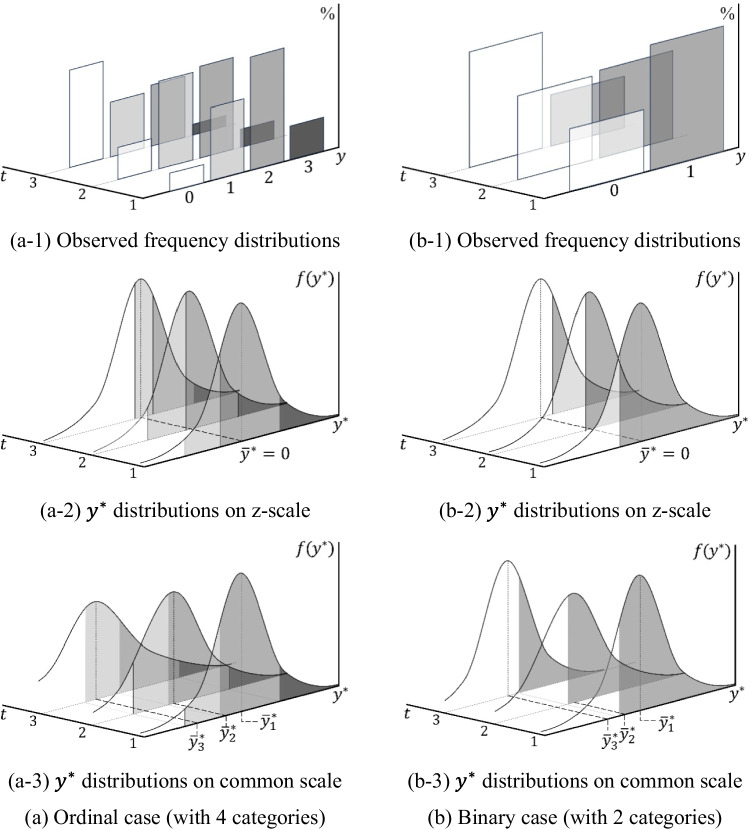
Process of deriving the sample distribution of ${y}^{*}$ for model estimation using the proportion information of observed $y$ when using ordinal and binary variables. Note. The observed frequency distributions are assumed to be obtained from the same underlying true population distribution as illustrated in Fig. 5; accordingly, the final distribution of ${y}^{*}$ on the common scale is expected to reproduce the population distribution in Fig. 5a

Nevertheless, as previously noted, the use of different observed scale references in the two cases precipitates differences in the distribution characteristics of ${y}^{*}$. Figure 6 visually depicts these differences. Both ordinal and binary LGMs transform the observed $y$ (Fig. 6a-1 and b-1) into a time-specific ${y}^{*}$ on the z-scale (Fig. 6a-2 and b-2) and, subsequently—by setting a common scale for all time points—follow the same procedure to calculate the mean and standard deviation of ${y}^{*}$ at each time point (Fig. 6a-3 and b-3). In the case of the ordinal LGM, the scale unit at each time point is defined by the intervals between the thresholds across time. Accordingly, the resulting ${\overline{y} }_{t}^{*}$ and $\sqrt{{s}_{t}^{*}}$ can provide appropriate information for estimating the population parameters ${\mu }_{t}^{*}$ and $\sqrt{{\sigma }_{t}^{*}}$. Figure 6a-3 shows that the distribution of ${y}^{*}$ on the common scale used for model estimation correctly reproduces the population-level distribution of ${y}^{*}$ in Fig. 5a, which generated the observed variables. In contrast, in the case of binary LGM, because the scale unit at each time point is determined solely by the location of the threshold at that moment and follows a specific pattern, the model fails to appropriately reflect the population-level variability that changes over time. Consequently, as shown in Fig. 6b-3, the computed ${\overline{y} }_{t}^{*}$ and $\sqrt{{s}_{t}^{*}}$ at each time point deviate from the values expected at the population level, and the distribution of ${y}^{*}$ on the common scale differs from the population-level distribution of ${y}^{*}$ illustrated in Fig. 5a. This leads to systematic bias in the estimation of ${\mu }_{t}^{*}$ and $\sqrt{{\sigma }_{t}^{*}}$, and inevitably undermines the estimation of growth factors.

Unlike ordinal LGMs, binary LGMs are limited by the nature of their observed scale references. This limitation results in a distribution of ${y}^{*}$ that deviates from what would be expected at the population level, potentially introducing bias into the estimation of all model parameters, including growth factors. In the context of categorical LGMs, which rely on transforming all observed categorical variables into latent response variables, responses observed through a binary scale are inherently insufficient for recovering complete information about the population distribution of ${y}^{*}$. That is, the distributional characteristics of ${y}^{*}$ defined through binary observed variables are likely to be inaccurate, thereby undermining the credibility of the estimated growth trajectories. This suggests that the use of binary variables in categorical LGMs may be an inappropriate modeling choice.

## An illustrative simulation study

This study builds on the preceding theoretical discussion regarding the impact of observed scale references in ordinal and binary LGMs and examines, through a simple simulation, how the estimation performance of categorical LGMs varies depending on the number of response categories. The simulation generates datasets based on the same underlying population distribution of ${y}^{*}$ but with varying numbers of response categories and compares the estimation performance of models across these conditions.

### Data generation

The example utilizes a basic linear growth model with intercept and slope factors across four time points and compares estimation results using observed variables with two to four categories. In the data generation process, one critical consideration was that all observed categorical data should be based on the same underlying population of ${y}^{*}$, with only the number of categories varying across conditions, as would be the case in real-world settings. To this end, continuous ${y}^{*}$ values were randomly generated from a normally distributed population. Then, categorical variables were created by converting each ${y}^{*}$ value into a corresponding category according to a set of predefined threshold values. This approach requires specifying the distributional characteristics of ${y}^{*}$ (i.e., means and covariances) and threshold parameters for each time point.

The means of the underlying ${y}^{*}$ at four time points were determined based on the mean slope (i.e., ${\alpha }_{10}$) and change pattern in proportion, following Eq. 2. Table 1 presents all corresponding parameter values. Two levels of the average slope were employed—large (${\alpha }_{10}=0.40$) and small (${\alpha }_{10}=0.10$)—based on the findings of Newsom and Smith (2020), who suggested that the magnitude of ${\alpha }_{10}$ may influence the estimation performance of categorical LGMs. The pattern in proportion changes was set according to the characteristic of categorical LGMs, where the observed response proportions vary depending on the size of the mean under the same threshold conditions. The pattern is classified into the following three conditions based on how the frequency distribution of the observed categorical variable changes over time: (1) from a positively skewed to a negatively skewed distribution, (2) from a positively skewed to a symmetric distribution, and (3) from a symmetric to a negatively skewed distribution. These conditions are designated as shift, increase, and decrease, respectively. This is because, for binary variables under these conditions, the standard deviation of ${y}^{*}$, $\sqrt{{s}_{t}^{*}}$, is expected to shift, increase, or decrease over time, regardless of the underlying population standard deviation, $\sqrt{{\sigma }_{t}^{*}}$, depending on the common scale setting.

**Table 1:** Population means of ${y}^{*}$ at each time point set according to conditions of patterns of proportion changes and slope means

Pattern	Slope mean	${\mu }_{1}^{*}$	${\mu }_{2}^{*}$	${\mu }_{3}^{*}$	${\mu }_{4}^{*}$
Shift	High	− 0.60	− 0.20	0.20	0.60
	Low	− 0.15	− 0.05	0.05	0.15
Increase	High	− 1.20	− 0.80	− 0.40	0.00
	Low	− 0.30	− 0.20	− 0.10	0.00
Decrease	High	0.00	0.40	0.80	1.20
	Low	0.00	0.10	0.20	0.30

Note. ${\mu }_{t}^{*}$ is the population mean of the ${y}^{*}$ distribution at time $t$

**Fig. 7:**
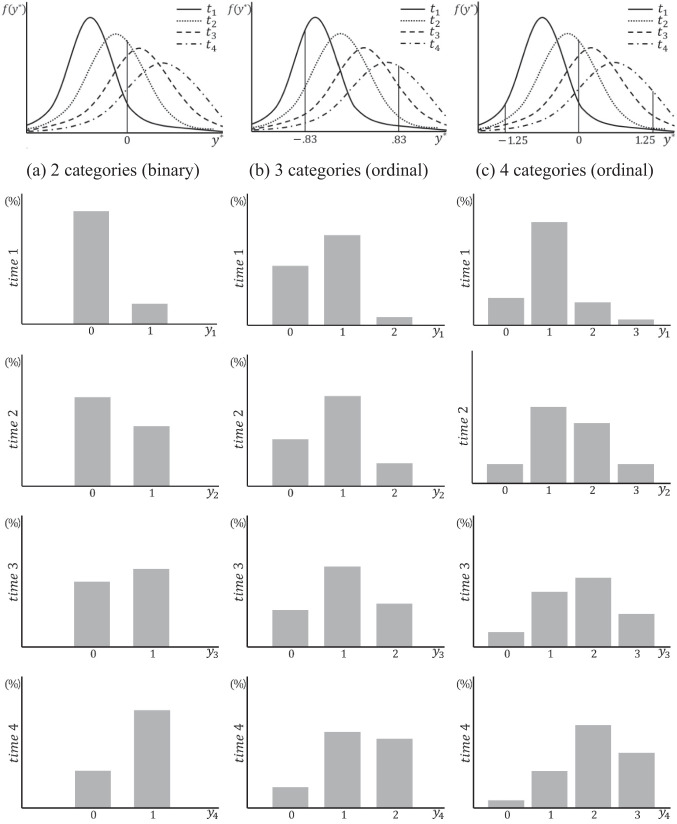
Examples of shift pattern condition across four time points by number of categories

Next, the covariance parameters of the underlying population ${y}^{*}$ were specified as factor covariances of ${\psi }_{00}=0.5$, ${\psi }_{11}=0.1$, and ${\psi }_{10}=0$, with error variances at each time point set as ${\theta }_{1}=0.5$, ${\theta }_{2}=0.6$, ${\theta }_{3}=0.9$, ${\theta }_{4}=1.4$, as derived from Eqs. 3 and 4. All values used in the covariance matrix setting were grounded in Muthén and Asparouhov (2002), reflecting the belief that in real-world scenarios, error variance increases over time because of various factors, such as maturation or fatigue effects. Hence, the standard deviation of ${y}^{*}$ in the population, $\sqrt{{\sigma }_{t}^{*}}$, was set to increase over time. Finally, the threshold parameters of the underlying population ${y}^{*}$ for each number of categories were specified as follows: for the two-category condition, ${\tau }_{1}=0$; for the three-category condition, ${\tau }_{1}=-0.83$ and ${\tau }_{2}=0.83$; and for the four-category condition, ${\tau }_{1}=-1.25$, ${\tau }_{2}=0$, and ${\tau }_{3}=1.25$. These were set following the symmetric threshold conditions provided by Rhemtulla et al. (2012).^2^ The thresholds interact with the previously specified means and, thereby, determine the pattern of change in proportion over time. Figure 7 visually presents some examples of the frequency distributions at all time points for the categorical data generated under these conditions.

Based on the specified means, covariances, and thresholds parameters, data regarding the continuous latent response variable ${y}^{*}$ were generated under three sample size conditions—specifically, two with small sizes ($N=100$ and $N=200$) and one with a large size ($N=\mathrm{1,000}$)—to enable a clearer comparison of results. Then, the generated continuous data at each time point were transformed into ordinal or binary variables using the threshold parameters defined per the number of categories. Through this process, a total of 1,000 datasets were generated for each of the 54 simulation conditions, which were defined by a combination of slope magnitude (two levels), pattern of change in proportion (three levels), number of categories (three levels), and sample size (three levels). All datasets were generated using R 4.2.1.

### Data analysis and evaluation criteria

For the generated datasets, categorical LGMs assuming a linear growth trajectory were estimated using Mplus 8.3 (Muthén & Muthén, 1998–2017). Weighted least squares with mean and variance adjusted (WLSMV) was selected for the estimation because it is known to be computationally faster than traditional WLS while maintaining relatively high accuracy and efficiency (Edwards, 2010; Nussbeck et al., 2006). The observed variables were scaled by applying the WLSMV-delta parameterization, in which the variance of ${y}^{*}$ at the first time point is fixed. This approach has been widely adopted in previous studies because of its ability to reflect the characteristics of categorical LGMs and provide interpretational advantages (Lim & Kim, 2024). Performance comparisons have focused on the estimated slope factor parameters of the growth model—namely, the factor mean (${\alpha }_{10}$) and factor variance (${\psi }_{11}$)—as these are typically the primary parameters of interest for researchers using LGMs. In addition, the estimated scale parameters (${\Delta }_{t}$), corresponding to the inverse of the standard deviation of ${y}^{*}$ at each time point (Muthén & Asparouhov, 2002), are reported because the performance differences between ordinal and binary LGM estimations may arise from distortions in the standard deviation of ${y}^{*}$, as explained previously. Note that ${\Delta }_{1}$ was fixed at 1 for the identification.

Estimation performance was evaluated using the following three criteria across conditions: estimation difficulties, relative bias (RB) of parameter estimates, and root mean square error (RMSE). Estimation difficulties refer to the proportion of replications under each condition that resulted in improper solutions or non-convergence (Newsom & Smith, 2020). The RB of an estimate refers to the proportional difference between the estimated parameter and the true population parameter, with values closer to zero indicating more accurate estimation. Per previous research (Curran et al., 1996), an RB of less than 5% is deemed to indicate trivial bias, an RB between 5 and 10% is regarded as indicative of moderate bias, and an RB exceeding 10% is considered to indicate substantial bias. The RMSE is a measure used to evaluate the variability of parameter estimates across replications, with values closer to zero indicating more stable estimation.

### Simulation results

Table 2 presents estimation difficulties under the four simulation conditions, revealing that estimation difficulties were significantly greater when employing binary rather than ordinal variables. This tendency was particularly pronounced when the slope was small and the sample size was limited, which indicates that estimation using binary LGMs is highly likely to precipitate non-convergence or improper solutions, thereby rendering the results unreliable. Considering that model misspecification is among the main causes of model non-convergence, the high estimation difficulties observed in the binary variable conditions can be attributed to distortions in the distributional characteristics of ${y}^{*}$ transformed from the generated categorical variables, making them unsuitable for model estimation. However, this issue seems to be mitigated to some extent when the sample size is sufficiently large, even with binary variables. Another notable finding was that, under the decrease pattern and large slope mean conditions, the estimation difficulties for the ordinal LGM with four categories were also very high at 13.7%. Given the parameters specified in this example, the proportion of responses at the negative extreme (i.e., responses of 0) becomes very small at all time points under this condition. Consequently, consistent with prior studies based on categorical CFA, this condition may have caused estimation issues due to the extremely skewed thresholds—namely, when a specific category shows a very low response rate across all indicator variables (Babakus et al., 1987; Lei, 2009; Rhemtulla et al., 2012). In other words, it is likely that model estimation was adversely affected under this condition because none of the indicators had sufficient response information for the extreme category.

**Table 2:** Estimation difficulties (%) for generated datasets

Pattern	Slope mean	$N$	Number of categories		
			2	3	4
Shift	High	100	40.7	0.0	0.0
		200	0.0	0.0	0.0
		1,000	0.0	0.0	0.0
	Low	100	63.0	0.0	0.0
		200	42.0	0.0	0.0
		1,000	3.2	0.0	0.0
Increase	High	100	47.7	0.0	0.0
		200	27.3	0.0	0.0
		1,000	0.0	0.0	0.0
	Low	100	58.5	0.0	0.0
		200	34.8	0.0	0.0
		1,000	0.0	0.0	0.0
Decrease	High	100	52.8	0.0	13.7
		200	32.7	0.0	0.0
		1,000	0.0	0.0	0.0
	Low	100	68.6	0.0	0.0
		200	54.4	0.0	0.0
		1,000	14.5	0.0	0.0

Tables 3, 4 and 5 present the estimation results for all model parameters. Table 3 presents the estimates for the slope mean and variance, as well as the scale parameter estimates, under the shift pattern condition. For the ordinal LGM (with three or four categories), all parameters were estimated accurately and stably, with RB values smaller than 5% and RMSE values close to 0. For the binary LGM, when the parameter for the slope mean was large, ${\alpha }_{10}$ demonstrated satisfactory estimation results, with RB values under 5% across all levels of sample sizes. However, ${\psi }_{11}$ was significantly biased in cases with small sample sizes (100 or 200), which is believed to be associated with the bias and instability observed in the estimation of scale parameters (i.e., $\Delta$ s). These parameters include information about the model’s standard deviation estimates (i.e., $\sqrt{{\widehat{\sigma }}^{*}}$) at each time point in these conditions. As presented in Eq. 3, $\sqrt{{\widehat{\sigma }}^{*}}$ is closely related to the estimation of ${\psi }_{11}$ in categorical LGMs. This suggests that, as the standard deviation estimate of ${y}^{*}$ becomes inaccurate in the binary LGM, the estimation of ${\psi }_{11}$ is also negatively affected. This tendency was more pronounced when the change in the response rates was minimal across time points (i.e., the slope mean was small). When the slope mean was small, the estimates of $\Delta$ s and ${\psi }_{11}$ in the binary LGM were increasingly biased as the sample size decreased. Furthermore, in this condition, the RB for ${\alpha }_{10}$ was 52.9% at $N=100$ and 24.7% at $N=200$; this result indicates that, unlike when the slope mean was large, the estimated ${\alpha }_{10}$ was significantly biased when the sample size was not sufficiently large.

**Table 3:** Parameter estimation results under shift pattern condition

Parameters	Slope mean											
	0.40 (Large)						0.10 (Small)					
	Number of categories						Number of categories					
	2		3		4		2		3		4	
	RB	RMSE	RB	RMSE	RB	RMSE	RB	RMSE	RB	RMSE	RB	RMSE
$$N=100$$												
Slope												
${\alpha }_{10}$	.050	.169	.000	.074	− .022	.066	.529	.294	.009	.061	− .001	.058
${\psi }_{11}$	1.936	2.608	.025	.080	− .045	.062	34.510	30.693	.045	.075	.044	.069
Scales												
$${\Delta }_{1}$$												
${\Delta }_{2}$	.030	.318	.015	.127	.023	.102	.165	.664	.007	.118	.004	.097
${\Delta }_{3}$	.104	.408	.027	.122	.036	.097	.492	.999	.013	.109	.008	.092
${\Delta }_{4}$	.139	.364	.031	.114	.043	.096	1.951	3.234	.008	.098	.006	.081
$$N=200$$												
Slope												
${\alpha }_{10}$	.022	.088	− .001	.051	− .010	.048	.427	.247	− .006	.041	− .007	.040
${\psi }_{11}$	.432	.199	− .010	.052	− .014	.046	31.811	19.884	.006	.052	− .020	.045
Scales												
$${\Delta }_{1}$$												
${\Delta }_{2}$	.004	.211	.005	.086	.008	.071	.061	.500	.006	.079	− .005	.066
${\Delta }_{3}$	.033	.259	.009	.080	.013	.066	.287	.738	.005	.075	.008	.062
${\Delta }_{4}$	.062	.243	.011	.075	.017	.062	1.096	2.079	.006	.069	− .005	.057
$$N=\mathrm{1,000}$$												
Slope												
${\alpha }_{10}$	.005	.036	.000	.022	.000	.020	.062	.045	.003	.020	− .004	.017
${\psi }_{11}$	.062	.049	.001	.024	− .004	.020	5.509	8.899	− .001	.022	.000	.020
Scales												
$${\Delta }_{1}$$												
${\Delta }_{2}$	.001	.096	.000	.039	.000	.033	.003	.293	.001	.037	.000	.030
${\Delta }_{3}$	.010	.112	.002	.036	.001	.030	.079	.378	.002	.033	.002	.026
${\Delta }_{4}$	.006	.095	.001	.033	.001	.028	.212	.565	− .003	.032	− .001	.026

Notes. ${\alpha }_{10}$ is the slope mean, ${\psi }_{11}$ is the slope variance, and ${\Delta }_{t}$ is the scale parameter at time $t$. RB is the relative bias, and RMSE is the root mean square error

**Table 4:** Parameter estimation results under the increase pattern condition

Parameters	Slope mean											
	0.40 (Large)						0.10 (Small)					
	Number of categories						Number of categories					
	2		3		4		2		3		4	
	RB	RMSE	RB	RMSE	RB	RMSE	RB	RMSE	RB	RMSE	RB	RMSE
$$N=100$$												
Slope												
${\alpha }_{10}$	− .040	.112	− .040	.066	− .064	.064	.010	.246	.012	.061	.003	.057
${\psi }_{11}$	.865	.544	− .106	.076	− .116	.061	28.167	16.886	.047	.074	.007	.062
Scales												
$${\Delta }_{1}$$												
${\Delta }_{2}$	.003	.197	.044	.140	.055	.118	.066	.540	.010	.119	.009	.101
${\Delta }_{3}$	.072	.307	.064	.139	.088	.122	.301	.764	.012	.108	.008	.089
${\Delta }_{4}$	.478	1.327	.086	.132	.111	.118	1.599	2.987	.022	.108	.017	.088
$$N=200$$												
Slope												
${\alpha }_{10}$	− .014	.069	− .018	.047	− .028	.045	− .369	.202	− .003	.042	− .016	.040
${\psi }_{11}$	.337	.164	− .038	.056	− .102	.044	21.082	13.450	.026	.052	− .007	.046
Scales												
$${\Delta }_{1}$$												
${\Delta }_{2}$	− .003	.135	.021	.101	.028	.081	− .014	.406	.004	.083	.004	.067
${\Delta }_{3}$	.024	.210	.030	.096	.044	.079	.135	.569	.005	.077	.008	.064
${\Delta }_{4}$	.196	.571	.036	.090	.051	.074	.746	1.794	.010	.073	.011	.060
$$N=\mathrm{1,000}$$												
Slope												
${\alpha }_{10}$	− .006	.024	− .003	.022	− .008	.020	− .045	.033	− .005	.020	− .002	.020
${\psi }_{11}$	.046	.050	− .016	.026	− .036	.022	.828	.387	.008	.022	.003	.020
Scales												
$${\Delta }_{1}$$												
${\Delta }_{2}$	.002	.060	.005	.047	.009	.037	− .007	.203	.000	.037	.001	.032
${\Delta }_{3}$	.008	.095	.008	.044	.012	.035	.028	.265	.000	.035	.000	.028
${\Delta }_{4}$	.038	.136	.010	.041	.014	.032	.111	.356	− .001	.032	.002	.026

Notes. ${\alpha }_{10}$ is the slope mean, ${\psi }_{11}$ is the slope variance, and ${\Delta }_{t}$ is the scale parameter at time $t$. RB is the relative bias, and RMSE is the root mean square error

**Table 5:** Parameter estimation results under the decrease pattern condition

Parameters	Slope mean											
	0.40 (Large)						0.10 (Small)					
	Number of categories						Number of categories					
	2		3		4		2		3		4	
	RB	RMSE	RB	RMSE	RB	RMSE	RB	RMSE	RB	RMSE	RB	RMSE
$$N=100$$												
Slope												
${\alpha }_{10}$	.556	1.132	− .016	.081	− .014	.073	.610	.304	.021	.061	− .001	.056
${\psi }_{11}$	17.003	12.660	.006	.077	.004	.068	25.695	21.715	.076	.076	.057	.066
Scales												
$${\Delta }_{1}$$												
${\Delta }_{2}$	.252	.824	.021	.125	.019	.099	.257	.949	.009	.120	− .001	.096
${\Delta }_{3}$	.491	1.059	.023	.114	.020	.095	.542	1.010	.012	.116	.005	.091
${\Delta }_{4}$	1.023	1.943	.037	.112	.028	.091	2.260	3.453	.020	.109	− .009	.087
$$N=200$$												
Slope												
${\alpha }_{10}$	.477	.840	.003	.060	− .004	.051	.868	.365	.004	.042	− .021	.040
${\psi }_{11}$	10.041	8.717	.057	.055	.032	.049	22.081	11.301	.045	.052	.014	.046
Scales												
$${\Delta }_{1}$$												
${\Delta }_{2}$	.065	.490	.003	.090	.003	.073	.174	.577	.005	.085	.004	.070
${\Delta }_{3}$	.163	.569	.005	.079	.006	.067	.480	.880	.006	.076	.003	.063
${\Delta }_{4}$	.264	.663	.006	.073	.009	.062	1.885	2.984	.010	.073	.008	.057
$$N=\mathrm{1,000}$$												
Slope												
${\alpha }_{10}$	.085	.173	.003	.024	.002	.022	.644	.280	.003	.020	.001	.017
${\psi }_{11}$	.599	.282	.009	.024	.003	.022	16.272	10.319	.004	.022	.006	.020
Scales												
$${\Delta }_{1}$$												
${\Delta }_{2}$	.008	.227	.001	.039	.001	.032	.047	.402	.002	.036	.001	.030
${\Delta }_{3}$	.023	.225	.001	.035	.001	.028	.192	.557	.001	.033	.000	.028
${\Delta }_{4}$	.033	.201	.000	.032	.000	.028	.616	1.311	− .001	.032	− .001	.026

Notes. ${\alpha }_{10}$ is the slope mean, ${\psi }_{11}$ is the slope variance, and ${\Delta }_{t}$ is the scale parameter at time $t$. RB is the relative bias, and RMSE is the root mean square error

Table 4 presents the estimation results for the model parameters under the increase pattern condition. For the ordinal LGM, ${\alpha }_{10}$ and ${\psi }_{11}$ were estimated accurately and stably, regardless of the size of the slope mean or the sample size. In the case of the binary LGM, when the sample size was small and the slope mean was small, the parameter estimates (particularly ${\psi }_{11}$) were severely biased, like the previous shift pattern condition. Overall, the estimation results under the increase pattern condition generally demonstrated smaller biases than the shift pattern condition. This can be understood as being a result of the standard deviation $\sqrt{{s}^{*}}$ in this condition following an increasing pattern over time, which mirrors the change in the population’s standard deviation $\sqrt{{\widehat{\sigma }}^{*}}$, thereby better reflecting the population characteristics. However, it should be noted that this improvement is only relative to the shift pattern condition, and the estimation results for the binary LGM in this condition should not be considered as fully reflecting the population characteristics.

Table 5 presents the estimation results for all model parameters under the decrease pattern condition. As with the previous results, the estimates of ${\alpha }_{10}$ and ${\psi }_{11}$ were accurate and stable for the ordinal LGM but significantly biased for the binary LGM. Notably, the overall estimation results for the binary LGM under this condition were evidently worse than those under the shift or increase pattern conditions. In the previous conditions, ${\alpha }_{10}$ exhibited no significant bias when the slope mean was large or when the sample size was sufficiently large. However, under the decrease pattern condition, when the sample size was large, the RB for ${\alpha }_{10}$ was 8.5%, indicating a moderate level of bias, and when the sample size was small, the RB was greater than 10%, indicating a severe bias. Moreover, in the condition with a small slope mean, the bias for ${\alpha }_{10}$ and ${\psi }_{11}$ was not sufficiently reduced even when the sample size was 1,000. One possible reason for the more severe biases under this condition is the binary LGM’s failure to capture the true pattern of the population. Specifically, while the population standard deviation ($\sqrt{{\sigma }^{*}}$) increased over time, the estimated standard deviation of ${y}^{*}$ ($\sqrt{{s}^{*}}$) in the binary LGM actually decreased. This mismatch between the model and the population likely led to inaccurate parameter estimates.

In sum, the binary LGM exhibited not only frequent estimation issues, such as non-convergence and improper solutions, but also poor accuracy and stability in estimating key parameters, including the slope mean and variance. In contrast, the ordinal LGM demonstrated accurate and stable performance across all conditions, even when the number of categories was as few as three—comparable to results with four categories. These findings suggest that the estimation problems observed in the binary LGM are not merely attributable to the smaller number of categories but might have instead stemmed from fundamental differences in the scaling and estimation processes between ordinal and binary LGMs.

## Discussion

This study primarily aimed to examine the adverse impact of employing binary observed variables on model estimation in categorical LGMs. In SEM-based models utilizing categorical data, binary variables have traditionally been treated as a special case of ordinal variables, with both types being regarded as categorical and subjected to the same estimation procedures (Agresti, 2018; Kamata & Bauer, 2008; Muthén & Asparouhov, 2002). However, a critical distinction exists between models employing ordinal versus binary variables: the choice of observed scale references used to set the metric of the latent response variable. Particularly in the context of LGM, the scale references applied to binary variables are more likely to hinder model estimation. Nevertheless, limited attention has been paid to the systematic negative consequences potentially arising from the utilization of binary variables during scale setting in categorical LGMs. This lack of prior discussion has led researchers to prematurely attribute the estimation differences between ordinal and binary LGMs solely to information loss due to fewer response categories in binary variables. To address this gap, this study theoretically investigated how differences in observed scale references influence the estimation of ordinal and binary LGMs and provided empirical evidence, through simulation studies, of the detrimental effects of scale referencing in binary LGM estimation.

The observed scale references (e.g., thresholds and standard deviations) are a key concept that determines the scale of the latent response variable derived from an observed variable. Differences in these references can alter the distributional characteristics of the latent response variable used in model estimation, such as its mean and standard deviation. The present study demonstrated how the scale reference of observed variables in categorical LGM governs the distributional properties of the latent response variable, and how these properties, in turn, adversely affect the estimation of binary LGMs—a process illustrated through a series of figures and equations. In ordinal LGMs, two thresholds are used as the observed scale references. This approach enables the latent response variable to accurately reflect the standard deviation of the underlying population distribution. In contrast, binary LGMs use the standard deviation of the binary variable as the observed scale reference. Consequently, the distribution of the transformed latent response variable may deviate substantially from the target population distribution, leading to biased estimates across the model.

The estimation issues associated with binary LGMs were also empirically demonstrated through simulation examples. The results revealed that, even when categorical data were generated from an identical population-level distribution, the estimation performance of LGMs was substantially influenced by whether the observed variables were binary or ordinal. In ordinal LGMs, all model estimates were accurate and stable regardless of sample size, the magnitude of the slope means, or the pattern of changes in standard deviations across time. This held true even when the ordinal variables had only three categories—just one more than binary variables. Moreover, increasing the number of categories from three to four within the ordinal condition only marginally improved estimation performance. In contrast, binary LGMs produced acceptable estimates only when specific conditions were met: a sufficiently large sample size, substantial mean changes over time, and a pattern of observed response proportions that appropriately reflected the population-level variation in standard deviations. However, such favorable conditions are rarely satisfied in typical empirical studies. Researchers typically have limited control over the magnitude of mean or standard deviation changes in the population over time. Thus, unlike ordinal LGMs, binary LGMs are likely to yield unreliable parameter estimates in most practical research settings. These findings suggest that using binary observed variables in LGMs—when the objective is to examine longitudinal changes in key constructs—may be problematic and potentially risky.

Although this study achieved its primary aim of theoretically and empirically demonstrating estimation issues in binary LGMs, some limitations should be noted. First, to clearly illustrate the differences in estimation performance between ordinal and binary LGMs, we deliberately employed a simple linear model with four time points and restricted the number of response categories to between two and four. However, as noted in the results, ordinal LGMs may also exhibit poor performance when the number of response categories is excessively large, as this can result in asymmetrical threshold locations at certain time points (Babakus et al., 1987; Lei, 2009; Rhemtulla et al., 2012). Therefore, future research should more comprehensively evaluate the performance of ordinal LGMs by varying the number of response categories and increasing the number of measurement occasions. Second, the simulations in this study employed the WLSMV-delta estimator that fixes the variance at the first time point. This approach is generally recommended for empirical studies due to its suitability for modeling heteroscedastic LGM structures and the ease of interpreting its parameter estimates (Lim & Kim, 2024). However, some previous studies have reported that alternative estimation methods—such as robust maximum likelihood (Newsom & Smith, 2020) or multilevel IRT-based approaches using Bayesian or Laplace estimation (Ye, 2016)—may outperform WLSMV in the context of binary LGMs. Thus, additional research is needed to determine whether alternative estimators can mitigate the systematic negative effects arising from scale-setting issues discussed in the present study.

Lastly, while not a limitation per se, it is necessary to acknowledge models that utilize categorical variables beyond LGM. The difference in the observed scale references applied to ordinal and binary variables occurs in not only LGM but also the estimation process of all SEM-based models employing categorical variables. As mentioned in the Introduction, in models such as CFA, where all observed variables are expected to have similar response proportions, the difference in observed scale references may have less impact on the estimation. Similar response proportions would result in similar scale unit lengths for all variables in the model, irrespective of whether they are sequentially or simultaneously determined—in the ordinal and binary cases, respectively. In either case, the standard deviations and means of the latent response variables are expected to be similar. That is, the difference in the observed scale references applied to ordinal and binary variables is unlikely to substantially alter the distribution of the latent response variables and, thus, may have minimal impact on estimation performance. However, even when using CFA, if the population-level homogeneity of variance across variables cannot be assumed, or if the observed data exhibit considerable differences in response proportions across variables, the resulting distributions of the latent response variables may differ significantly between the ordinal and binary cases. This suggests that, depending on the nature of the variables or the characteristics of the data, estimation in binary CFA may also deteriorate substantially, as in binary LGM. Therefore, further investigation is necessary to determine how observed scale references affect model estimation in binary CFA, particularly when the data exhibit substantially different proportions across observed variables. Moreover, such effects warrant more careful scrutiny in the context of other SEM-based models that frequently employ binary variables, such as latent class analysis (Lazarsfeld, 1950; Lazarsfeld & Henry, 1968).

Despite these limitations, this study significantly contributes to the literature by elucidating the performance disparities in the estimation of ordinal and binary LGMs, particularly emphasizing the influence of observed scale references in categorical variables on model estimation—a topic that has not been addressed thus far. By providing both theoretical insights and empirical evidence, this study thoroughly discusses the potential negative impacts of using binary observed variables in LGM estimation. Its findings suggest that, in the LGM context, employing ordinal variables is preferable to binary variables for more accurate and stable model estimates. This study will, hopefully, enable future researchers to better recognize the limitations of the information provided by binary observed variables and make more informed decisions in selecting the most appropriate data type for their models.

## Data Availability

The data used in this study are simulated and are available at OSF: https://osf.io/e4bgh/?view_only=bf799faa8d6d48f08561b0f3be08274b

## Footnotes

1 In categorical LGMs, the distributional characteristics of ${y}^{*}$ are defined by establishing a common scale including not only the mean and standard deviation but also covariance (or correlation) information (Lim & Kim, 2024). However, we focus on the mean and standard deviation of ${y}^{*}$ at each time point to more clearly delineate the distributional differences in latent response variables caused by scale differences.

2 In this study, the thresholds were randomly selected in order to generate various patterns of category proportions through combinations with mean-change conditions. In categorical LGMs, however, the means of the underlying ${y}^{*}$ change over time; therefore, even when applying these conditions, the thresholds for all observed indicators are not uniformly symmetric around the means, as reported by Rhemtulla et al. (2012) (see also Lim & Kim, 2024).

## Code availability

The Mplus codes used in this study are available at the same OSF repository: https://osf.io/e4bgh/?view_only=bf799faa8d6d48f08561b0f3be08274b
